# Irisin: circulating levels in serum and its relation to gonadal axis

**DOI:** 10.1007/s12020-022-02981-5

**Published:** 2022-01-17

**Authors:** Yunyao Luo, Xiaoyong Qiao, Liangzhi Xu, Guoning Huang

**Affiliations:** 1 Chongqing Key Laboratory of Human Embryo Engineering, Chongqing Reproduction and Genetics Institute, Chongqing Health Center for women and Children, No.64 Jin Tang Street, Yu Zhong District, Chongqing, 400013 China; 2 Reproductive Endocrinology and Regulation Laboratory West China Second University Hospital, Sichuan University, Chengdu, China; 3 Department of Obstetrics and Gynecology, West China Second University Hospital, Sichuan University, Chengdu, People’s Republic of China

**Keywords:** Irisin, Polycystic ovary syndrome, HPG axis, Reproduction, Fertility

## Abstract

Irisin is an exercise-induced myokine/adipokine in mice and humans that plays an important role in ‘browning’ of white adipose tissue and has shown great potential as a treatment for some metabolic diseases, such as obesity, insulin resistance, and inflammation. The circulating irisin level is reported to be associated with exercise, obesity, diet, diseases, and exposure to different pharmacological agents. Several studies have attempted to characterize the role of irisin in PCOS and other reproductive diseases, but contradictory results have been reported. Our previous study showed that irisin may serve further functions in folliculogenesis and fertility. In this review, we present the current knowledge on the physiology of irisin and its role in gonadal axis. Firstly, we describe irisin circulating levels and speculate on the potential mechanisms involved in irisin secretion and regulation. Then, we focus on the irisin levels in PCOS, and explore the relationships between, BMI, insulin resistance, and hyperandrogenism. Finally, we present the results from animal interventional studies and in vitro experiments to investigate the relationship between irisin and gonadal axis, indicating its novel effects on reproduction and fertility.

## Introduction

Irisin is a novel cytokine that is mainly secreted from skeletal muscle and was discovered by Bostrom in 2012 [1]. It is cleaved from its precursor, fibronectin type III domain containing 5 (FNDC5), and secreted during exercise, adrenergic stimulation, or exposure to cold environments [2]. Through binding to unknown receptors, irisin is proposed to induce ‘browning’ of white adipose tissue (WAT) [2, 3]. Studies have reported that irisin participates in energy expenditure, glucose uptake, and glycogenolysis by inhibiting gluconeogenesis, fat formation, and lipid accumulation and that it has potentially positive effects on glucose homeostasis and insulin sensitivity [2, 4, 5]. Therefore, based on these positive effects on metabolism, irisin is becoming a potential target for the treatment of metabolic diseases [6].

Reproductive health is closely related to the endocrine function of adipose tissue. Poor nutrition and low body mass are associated with the occurrence of infertility, whereas obesity and overweight also have adverse effects on fertility [7]. The impact of obesity on reproduction function, especially ovulatory disorders, are mainly attributable to endocrine mechanisms, which interfere with neuroendocrine and ovarian functions, and reduce the ovulation omeostatic. In men and women, body mass index (BMI) is reported to be negatively correlated with reproduction and fertility rate [8–10]. Obesity also has a negative impact on the outcomes of assisted reproductive technology (ART); the number of retrieved oocytes, ongoing pregnancy rate, and live birth rate are decreased in obesity [11–13]. Reproduction and fertility are closely linked to glucose and lipid metabolism. Hyperinsulinemia and insulin resistance (IR) interfere with follicle development and maturation, which can cause follicular dysplasia, ovulatory disorders, luteal phase defects, and poor fertility [14]. As irisin was discovered in components of the central nervous system (such as the brain and cerebrospinal fluid) and in reproductive organs (such as the seminiferous tubule, Leydig cells and epididymis) [15], its potential effects on reproduction have come into focus. Considering the positive effects of irisin on energy expenditure and glucose and lipid metabolism, irisin is likely to participate in reproductive health. Our previous study showed that irisin protein-knockout mice had disordered sex hormone metabolism and a significant decrease in fertility [16]. These findings not only showed the potential of irisin as a biochemical marker of many reproduction-related diseases but also led us to hypothesize that irisin might be directly involved in reproductive metabolism.

Some studies have focused on the effects of irisin on the secretion of sex hormones and the development of the gonads, but these studies have great heterogeneity, and some of the results need to be further verified. Therefore, the aim of this review is to summarize the currently existing evidence linking irisin with reproductive metabolism and to initially discuss the effects of irisin on reproduction and fertility.

## The secretion and regulation of irisin levels in different life phases

In rodents and humans, several factors were found to affect the physiological levels of irisin in the body, including exercise, obesity, diet, diseases, and exposure to different pharmacological agents [17–21]. Whether there is a difference in irisin levels between sexes is still unknown. Data from some research showed that the content of irisin in girls was higher than that in boys [22] and higher in women than in men [23]; however, Zugel [24] and Scalzo [25] reported that irisin concentrations at rest showed no difference in between sexes in adults. At present, the reported circulating levels of irisin seem to differ greatly even in the same species, with reported concentrations in humans ranging from 0.01 ng/ml to 2000 ng/ml [26–30].

Many studies have investigated the effects of exercise on irisin secretion and have reported contradictory results. For example, some animal and human studies have shown an increase in circulating levels of irisin after exercise. Irisin levels were reported to be significantly higher in people who exercise regularly than in sedentary people [31–34]. However, a few studies have not found a clear positive or negative association between these factors [35–37]. The discrepancy in results can be explained by the type of exercises conducted in the studies; for instance, irisin concentrations were significantly increased in strenuous exercise studies but not in endurance training studies [38, 39]. Interestingly, Hernandaz et al. reported that the level of irisin in newborns who were delivered vaginally was significantly higher than that in newborns delivered via cesarean section [40]. Since the levels of irisin in mothers were comparable between the two groups, the result could be partly explained by exercise. During vaginal delivery, fetal adaptive rotation is accompanied by changes in body pressure, which is similar to the effect of exercise, resulting in increased irisin levels.

In newborns, the expression of FNDC5 is very low, as is the level of circulating irisin, most of which comes from the mother through the placental barrier [40, 41]. Wrann et al. reported that the expression of FNDC5 in the brains of mice increased gradually from the 0th day to the 20th day after birth [42]. Although that study was not focused on the change of irisin level in circulation, the increased expression of its precursor protein implied that the secretion pattern of irisin changed from ‘maternal inheritance’ to ‘autonomous secretion’ and then remained relatively stable. The secretion of irisin is variable in different life phases (such as puberty, the child-bearing period or menopause) and different stages (such as during pregnancy and around childbirth), which might be attributed to fluctuations in hormone levels. During the menstrual cycle, cyclical patterns of irisin levels could be observed, including that the circulating irisin in luteal phase increased by approximately 25% compared with that in follicular phase but gradually decreased along with the regression of the corpus luteum and returned to the baseline level in the early follicular phase [43]. In pubertal children and pregnant women, irisin levels increased significantly with the activation of the HPO axis [44], and irisin levels were positively correlated with gestational age during pregnancy [43]. The mechanism of the increase in irisin, which may be related to large changes in sex hormones (mainly estrogen and progesterone), is not clear. In women aged 41–82 years old, irisin levels decreased with age [18, 45–47], which might be explained by changes in estrogen levels. For example, a decrease in estrogen levels was accompanied by a significant decrease in irisin levels in menopausal women aged 40–56 years old. Certainly, the loss of muscle and bone mass may be one of the reasons for the decline in irisin in menopausal women. Interestingly, our co-workers have showed irisin has positive effects on bone metabolism [48, 49]. The decrease of irisin level in menopausal women may in turn aggravate the development of osteoporosis. These effects were consistent with our previous study showing irisin could ameliorate bone loss in ovariectomized mice [50].

To date, the mechanism of the dynamic changes in irisin levels remains unknown, and we speculate that the fluctuation of irisin may represent a self-protective metabolic mechanism. For instance, insulin resistance always increases physiologically during adolescence and pregnancy, which stimulates the secretion of irisin to maintain glucose and lipid homeostasis [18, 51]. In addition, a higher level of irisin can inhibit oxidative stress and inflammation, which is important to the maintenance of pregnancy [52–55].

## Irisin and PCOS

Polycystic ovary syndrome (PCOS), a common endocrinopathy affecting 5–10% of women of reproductive age [56]. The Rotterdam diagnostic criteria for PCOS are now internationally endorsed and are based on two of three features: oligo- or anovulation, hyperandrogenism (clinical or biochemical), and polycystic ovaries [56]. Insulin resistance (IR) and hyperinsulinemia play an important role in the pathophysiology and metabolic manifestations of PCOS, independent of but exacerbated by obesity [56, 57]. To date, data on circulating irisin levels between women with PCOS and controls have been derived from case-control studies, which have been inconclusive. Most studies have reported higher irisin levels in women with PCOS than in controls [58–61]. Ovarian drilling of polycystic ovaries results in a significant decrease in serum irisin levels [62]. However, some other studies reported similar [63, 64] or lower [65] circulating irisin levels in women with PCOS than in controls.

It has been reported that some patients with PCOS can resume ovulation after weight loss, indicating the adverse effects of obesity on PCOS [66]. Many studies have demonstrated a positive relationship between circulating irisin levels and BMI in healthy people as well as in PCOS patients [26, 67]. In PCOS patients, irisin concentrations were higher in overweight and obese women than that in women with normal weight [59, 63, 68]. Weight loss led to a significant decrease in circulating irisin (15%), whereas weight regain returned irisin levels to baseline [59]. Therefore, the current controversial results of irisin level in PCOS may be related to the different baseline BMI level. The elevated irisin level may be a feedback mechanism to maintain metabolic balance in PCOS patients. Besides, it is also possible that there is irisin resistance in PCOS, similar to the insulin and leptin resistance observed in obesity and type 2 diabetes (T2DM) [43, 69, 70]. It should be highlighted that women with PCOS had a higher BMI than controls in most studies. In those patients, the increase of fat mass may induce the rising secretion of irisin, although these speculations remain to be elucidated [67].

Exercise and weight loss are well known to improve insulin sensitivity and prevent obesity and subsequent metabolic syndrome. As an exercise-induce cytokine, irisin, is reported to have multiple potential positive effects on glucose homeostasis and insulin sensitivity, such as promoting energy expenditure, glucose uptake, and glycogenolysis and reducing gluconeogenesis, adipogenesis, and lipid accumulation [2, 4, 5]. Although the etiology of PCOS remains unclear, IR plays an important role in its pathogenesis. Thus, the relationship between irisin and IR has become an interesting but controversial topic. Foda AA et al. [62] and Li et al. [59] indicated that circulating irisin was positively correlated with IR in PCOS women. After metformin treatment, the IR was improved in PCOS, and irisin level was reduced [59, 61]. On the other hand, Mareno et al. showed a negative relationship between irisin level and IR [18], whereas Choi [71] and Liu [19] reported no significant correlation between them.

In order to identify the role of irisin in IR, researchers focused on the relationship between irisin and insulin. In healthy people and patients with early diabetes, irisin has generally been negatively associated with insulin levels [18, 72]. However, with the decrease in insulin sensitivity, this relationship becomes positive; for example, irisin levels have been reported to be higher in individuals with diabetes mellitus [73, 74]. Animal studies also have shown that irisin may promote insulin secretion [75]. Therefore, irisin and insulin level showed dynamic fluctuation during changes in glucose homeostasis; however, the causal relationship between them remains unclear. Future research should focus on the effect of irisin on insulin secretion and signaling and vice versa and on possible interactions between the two pathways that might affect glucose homeostasis.

In addition, researchers have discovered relationships between irisin and androgen levels [63, 68], bone metabolism [64], and metabolic syndrome [63] in women with PCOS and have observed some meaningful outcomes. Hyperandrogenism is an endocrinological disorder in women with PCOS. Some studies have reported there was a positive correction between irisin and androgen or free androgen index in PCOS women [68]. Because hyperandrogenism is an important inducer of IR, the relationship of irsin to hyperandrogenism and IR needs further investigation.

Altogether, the contradictory results related to irisin in PCOS may be attributed to the heterogeneity of the research. On the other hand, it may also embody that the body has experienced a decompensation period. The initial endocrine disorder in PCOS patients could stimulate more irisin secretion to maintain the counterbalance of metabolism. Along with the further aggravation of metabolic abnormalities that go far beyond the body’s ability to compensate, a decrease in circulating irisin levels will occur. This is similar to the pattern of insulin levels in T2DM, which are elevated in the prediabetic state and reduced at an advanced stage of disease [59, 76].

In summary, the present review reported irisin may represent a novel PCOS biomarker and might directly contribute to the etiology of PCOS or indirectly affect the development of disordered metabolism (such as obesity, hyperandrogenism, impaired glucose tolerance, and insulin resistance) in PCOS [58]. The physiology and pathology of irisin in the development of PCOS remain to be further elucidated.

## Irisin and gonadal axis

The incidence of menstrual disorders in the general female population is approximately 5% [77], while it is as high as 12–79% of athletes [78–80]. Intense exercise is potentially harmful to the health of the female body, especially to reproductive function. Athletic menstrual cycle irregularities (AMI) are characterized by delayed menstruation, luteal phase defects, anovulation, hypomenorrhea and amenorrhea. The menstrual cycle can be gradually restored by reduced training intensity, and pregnancy can be achieved after stopping training. Fu et al. reported that the prolactin level in athletes with amenorrhea was significantly lower than that in athletes with normal menstrual cycles, while the levels of sex hormone binding globulin and growth hormone were significantly higher in the former group than in the latter, indicating that excessive exercise may interfere with the gonadal axis and influence hormone secretion, resulting in disordered reproductive function [81–83].

The hypothalamic-pituitary-ovarian (HPO) axis in females and the hypothalamic-pituitary-testicular (HPT) axis in males are the gonadal axes, which include hypothalamic neurons and their secreted gonadotropin-releasing hormone (GnRH), pituitary gonadotropin cells, and their synthetic follicle stimulating hormone (FSH) and luteinizing hormone (LH), estrogen (mainly estradiol) and progesterone (P) and testosterone (T). They interact with each other to constitute a complete and coordinated network that plays a crucial role in the reproductive system [84, 85].

To elucidate the possible mechanism of AMI, researchers have proposed the “body composition hypothesis”, “stress hypothesis” and “energy availability hypothesis” [86, 87]. At present, the “available energy hypothesis” proposed by Loucks et al. is generally accepted; this hypothesis holds that the imbalance between energy intake and energy expenditure is an important cause of menstrual disorders and that the inhibition of reproductive function is an adaptive response to reduced energy expenditure [88]. Many factors involved in regulating energy metabolism regulate reproductive health as well. For instance, insufficient energy intake reduced ghrelin production, which may inhibit reproductive functions by controlling GnRH, LH, and E_2_ release [89, 90]. In addition, leptin and triiodothyronine have been reported to have positive effects on reproduction [91–95]. As mentioned above, there are significant changes in circulating irisin levels in puberty, pregnancy, and the postpartum period, when the functions of the HPG axis are active. However, the relationship between irisin and the HPG axis is still unknown. This section aims to summarize the effects of irisin on the development of the HPG axis and steroid hormone secretion to further explore the potential role of irisin in reproductive health.

## The relationship of irisin to GnRH and gonadotropin

As important nerve centers, the hypothalamus and pituitary regulate downstream organs and hormone production through GnRH and gonadotropin, respectively. FNDC5 is mainly expressed in the proximal pars distalis (PPD) of the pituitary [96]. The existing data showed that irisin has positive effects on pituitary functions. In vivo, irisin could promote the secretion of FSH and LH in female rats [97, 98]. The female mice lacking irisin, showed lower concentrations of FSH and LH than those in wild-type mice [16]. Besides, in vitro, irisin treatment increased the expression of FSH and LH in the pituitary cells by improving the stability of transcription [96]. These results indicate that irisin may play a GnRH-like role in the HPG axis.

However, irisin showed opposing effects on gonadotropins when in combination with GnRH. For example, Ulker et al. showed that irisin exposure reduced the expression of GnRH in the hypothalamus, and delayed the onset of puberty (including significantly delayed vaginal opening time and estrous cycle) in female rats [98]. Besides, in vitro experiments, GnRH could promote the secretion of FSH and LH by pituitary cells when administered singlely, while the stimulating effects could be inhibited by irisin treatment in combination [96, 99]. According to the present results, we assumed that there may be two reasons for the negative effects of irisin on GnRH. First, irisin could promote the secretion of FSH and LH, inhibiting the release of GnRH through negative feedback. Second, we speculate that the effect of irisin is similar to that of gonadotropin-releasing hormone agonist (GnRH-a). The specific receptor of irisin in pituitary gonadotropin-secreting cells has thus far not been found. Therefore, irisin may bind to GnRH receptors on the cell surface, followed by a decrease in the number of available receptors and an increase in receptor internalization, resulting in pituitary desensitization, which inhibits the release of gonadotropins. The mechanism is similar to the process of pituitary suppression used in assisted reproductive technology [100, 101].

Therefore, irisin could promote the expression of FSH and LH, and on the one hand, irisin could compete with GnRH to inhibit the secretion of FSH and LH. These dual effects of irisin occur at the same time and interact with each other, and changes in circulating hormone levels are achieved when one activity is dominant.

## Irisin and ovarian/testicular and sex hormones

The ovary and testis, as downstream organs, are directly regulated by gonadotropin. The present data suggest that the interaction of irisin with FSH and LH may affect the development of the sexual gland. For example, irisin can promote the development of convoluted seminiferous tubules and the motility of germ cells in male rats [98]. FNDC5, the precursor protein of irisin, can promote the development of mouse ovaries, as reported by Bastu [97] et al., showing a significant increase in the number of primary and secondary follicles. Consistent with their results, our previous study showed that FNDC5-knockout mice had a decreased number of antral follicles. In contrast, some studies have reported that irisin has negative effects on ovaries and testes, significantly reducing the number of vegetative cells and Leydig cells, sperm density and mobility in male rats under irisin exposure [102], and causing a decrease in the number of primary follicles and a significant increase in ovarian fibrosis in female mice [98]. It has been reported that the physiological effect of irisin is concentration-dependent, which can partly explain the different or even opposite effects of irisin in metabolism. Irisin can promote the expression of LH in the pituitary gland of tilapia at a concentration of 1 nM, while the lowest concentration that promotes the expression of FSH is 10 nM [96]. In porcine ovarian granulosa cells, irisin at a low concentration (50 ng/ml) could promote progesterone secretion, while a high concentration (150 ng/ml) could inhibit the production of progesterone [103]. In addition, the effect of irisin is time-dependent. Wagner [104] et al. reported that long-term (>7 days) treatment with a supraphysiological dose (100–1000 ng/ml) of irisin could inhibit the expression of follicle stimulating hormone receptor (FSHR), while a physiological dose (10–100 ng/ml) of irisin or short-term (48 h) treatment had no significant effect on the expression of FSHR. Certainly, the use of different species and experimental protocols lead to high heterogeneity as well.

In terms of reproduction, nitric oxide (NO) can promote the meiosis of oocytes and the development of follicles and shows dual effects on follicular atresia [105, 106]. Interestingly, recent studies have reported the relationship between irisin and NO in the ovary. Bastu et al. reported that treatment with FNDC5 can promote the expression of nitric oxide synthase (NOS) in mouse ovaries [97] while significantly decreasing the expression of NO under irisin exposure in porcine ovarian granulosa cells [103]. The possible reason for the discrepancy is that NO can both promote and inhibit apoptosis. Moreover, the expression of NO is also affected by the complex environment in vivo; the atresia of follicles is regulated by many pathways, and the secretion of NO is affected by many factors. In short, there is a lack of evidence related to the direct effect of irisin on gonadal development and germ cell functions.

Sex steroids, including androgens, estrogens, and progesterone, are known to have widespread physiological actions in the reproductive system [107]. The present data showed a positive relationship between irisin and E_2_ levels [46, 98, 99], which may be related to the effect of irisin on FSH and LH. In addition, the effect of irisin on E_2_ synthesis could be due to irisin affecting ovarian aromatase activity. Our previous studies have confirmed that irisin can directly promote estrogen secretion by promoting the expression of CYP19A1, which is the rate-limiting enzyme of estrogen synthesis [16, 108]. To date, limited studies have described the relationship between irisin and progesterone. Our study initially reported an elevated progesterone level in FNDC5-knockout mice, which may be due to the downregulation of Akr1c18 gene expression, which in turn is mainly related to the removal of progesterone from the body. The results of irisin and androgen studies are inconsistent. Some studies observed a positive correlation between circulating irisin and testosterone levels [102, 109], while others showed a negative relationship between irisin and testosterone levels [59, 68] or no significant correlation [46].

## The other effects of irisin

Abnormal reproductive function eventually leads to decreased fertility and even infertility. The present data suggest that the infertility rate in China is between 12% and 15%; that is, more than 50 million couples are infertile, and infertility has become a common and serious public health problem [110, 111]. Reproduction and fertility are closely related to energy metabolism and the endocrine function of adipose tissue. Obesity or insufficient fat content will adversely affect fertility [112]. As regulators of energy expenditure, leptin and insulin contribute to reproductive health as well. For example, leptin participates in the regulation of energy, and its deficiency reduces endometrial receptivity, while insulin is a regulator of glucose and lipid metabolism, as well as endometrial receptivity [113, 114]. In addition, endometrial receptivity is significantly decreased in obesity [115]. Li et al. demonstrated that irisin may improve the receptivity of the endometrium by promoting the expression of leukemia inhibitory factor and integrin αvβ3 [116]. In addition, reproductive function is regulated by many other metabolic factors, such as the growth hormone axis (GH/IGFs), which is very important in gonadal development and fertility, including promoting oocyte maturation, granulosa cell proliferation, differentiation, and steroid hormone synthesis [117, 118]. Energy metabolism directly affects the secretion of GH and IGF-I, and some studies have reported that the levels of FNDC5 and/or irisin in circulation are positively correlated with the activity of the GH/IGF-I axis [46, 119, 120]. Therefore, irisin may have an indirect effect on fertility through the action of the growth hormone axis. Our previous study initially confirmed the effect of irisin on fertility in mice. In that study, irisin-deficient mice showed poor fertility, which was mainly characterized by significant decreases in the birth rate, average litter size per cage, the survival rate of newborn mice, and a significantly prolonged average time to litter production [76]. Due to the lack of available evidence, the specific effects and mechanisms of irisin on fertility are unclear, and more in-depth research is required to develop the field.

## Conclusion

Irisin is a novel myokine/adipokine that is implicated in the regulation of a variety of endocrine and metabolic functions, such as promoting WAT browning and glucose homeostasis. Irisin is appealing, with the potential to bridge our knowledge gaps related to the connection between exercise and beneficial effects on metabolic diseases. The circulating irisin level is likely related to sex, age, and physiological status. Clinical studies have indicated an association between circulating levels of irisin and PCOS. Interactions between irisin, BMI, insulin, androgens, or other hormones that are involved in the endocrine metabolic abnormalities in PCOS should be researched in the future. Many studies have underscored the positive role of irisin in reproductive health. Through its functions in the HPG axis, irisin contributes to the development of the ovary and testis and the secretion of steroids. Therefore, further research is required to confirm the relationship between irisin and other myokines and their causal relations. One crucial further step is to identify the specific receptor of irisin in the ovary and testis to characterize its mode of action, laying a solid foundation for the development of drugs for the treatment of various metabolic disorders and declining fertility.
